# Supplementary material for: KIF1A-driven entry of degradative lysosomes into the axon is developmentally regulated by the microtubule environment at the axon initial segment
Source: bioRxiv. 2026 Oct 30:2026.10.29.762862. Preprint. [Version 1] doi: 10.64898/2026.10.29.762862 (PMC13734103; doi:10.64898/2026.10.29.762862)
Supplement: Supplementary file 1 [file NIHPP2026.10.29.762862v1-supplement-6.pdf]

bioRxiv preprint doi: https://doi.org/10.64898/2026.10.29.762862; this version posted October 30, 2026. The copyright holder for this preprint (which was not certified by peer review) is the author/funder, who has granted bioRxiv a license to display the preprint in perpetuity. It is made available under a CC-BY 4.0 International license.

Supplementary material

Supplementary Figures 1-7

Supplementary Video 1. mNG-KIF1A and SiR-Lys movement through DIV3 proximal axon
Supplementary Video 2. mNG-KIF1A and SiR-Lys movement through DIV7 AIS
Supplementary Video 3. Comparison of LAMP1-mNG and SiR-Lys co-movement
Supplementary Video 4. Effect of AnkG/EB shRNA on mNG-KIF1A and SiR-Lys co-movement
Supplementary Video 5. Effect of T258M mutation on mNG-KIF1A and SiR-Lys co-movement

bioRxiv preprint doi: https://doi.org/10.64898/2026.10.29.762862; this version posted October 30, 2026. The copyright holder for this preprint (which was not certified by peer review) is the author/funder, who has granted bioRxiv a license to display the preprint in perpetuity. It is made available under a CC-BY 4.0 International license.

Figure S1

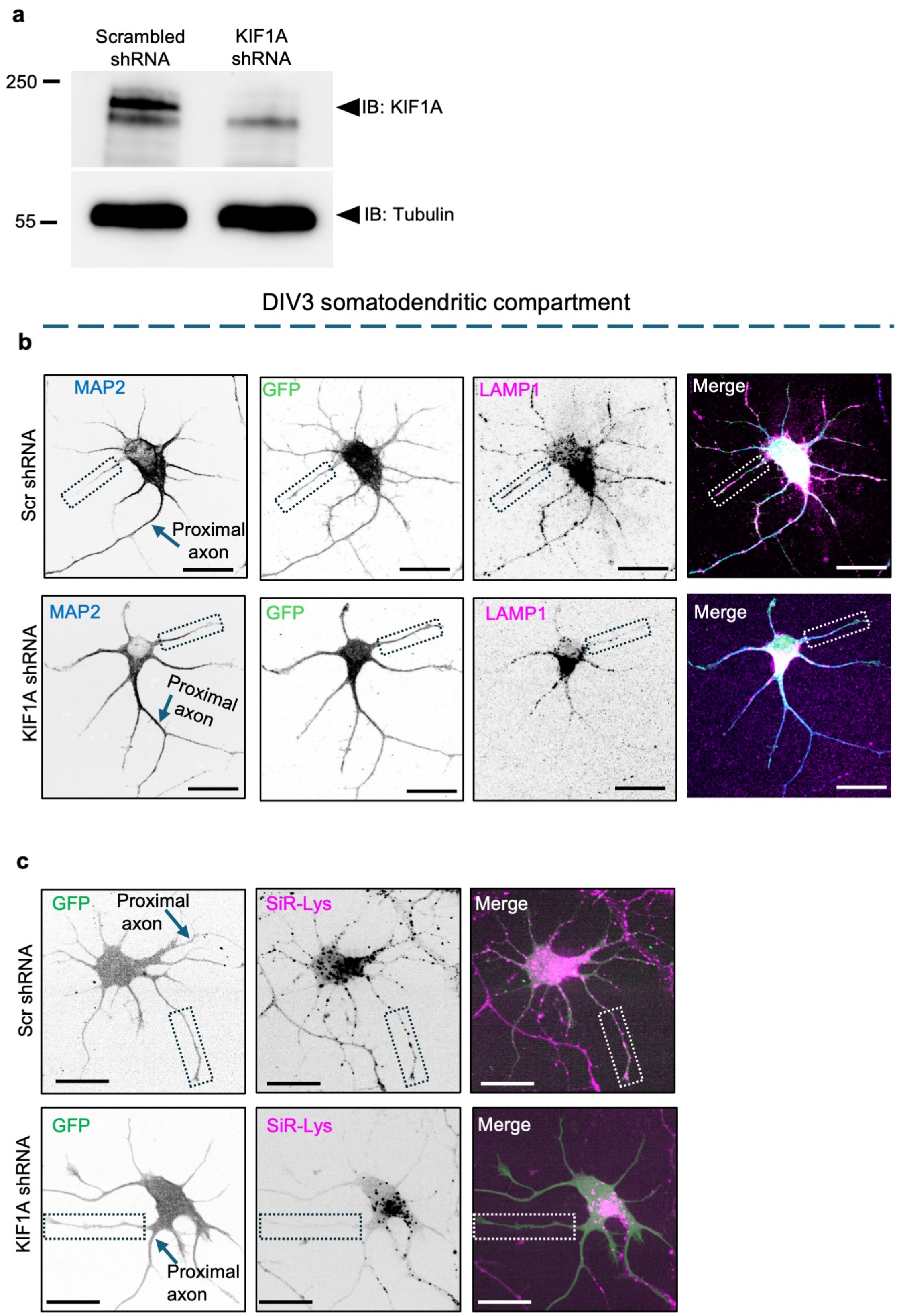

bioRxiv preprint doi: https://doi.org/10.64898/2026.10.29.762862; this version posted October 30, 2026. The copyright holder for this preprint (which was not certified by peer review) is the author/funder, who has granted bioRxiv a license to display the preprint in perpetuity. It is made available under a CC-BY 4.0 International license.

Figure S1. KIF1A depletion reduces lysosomal vesicle localization in the dendrites and axons of immature rat hippocampal neurons

a, Immunoblot showing depletion of KIF1A in rat hippocampal neurons infected with lentivirus encoding KIF1A shRNA. Tubulin was used as a loading control. Molecular masses are indicated in kilodaltons.

b, Confocal fluorescence microscopy of the somatodendritic compartment of DIV3 rat hippocampal neurons expressing Scr shRNA or KIF1A shRNA along with GFP, fixed, permeabilized, and immunostained for MAP2 and LAMP1. The dashed outlines indicate the dendrite tips shown in Fig. 1b. Single-channel images are shown in grayscale, and merged images in color with MAP2 in blue, GFP in green, and LAMP1 in magenta. Scale bars: 20 μm.

c, Confocal live-cell imaging of the somatodendritic compartment of DIV3 rat hippocampal neurons expressing Scr shRNA or KIF1A shRNA along with GFP and stained with SiR-Lys. The dashed outlines indicate the dendrite tips shown in Fig. 1f. Single-channel images are shown in grayscale, and merged images in color with GFP in green and SiR-Lys in magenta. Scale bars: 20 μm.

bioRxiv preprint doi: https://doi.org/10.64898/2026.10.29.762862; this version posted October 30, 2026. The copyright holder for this preprint (which was not certified by peer review) is the author/funder, who has granted bioRxiv a license to display the preprint in perpetuity. It is made available under a CC-BY 4.0 International license.

Figure S2

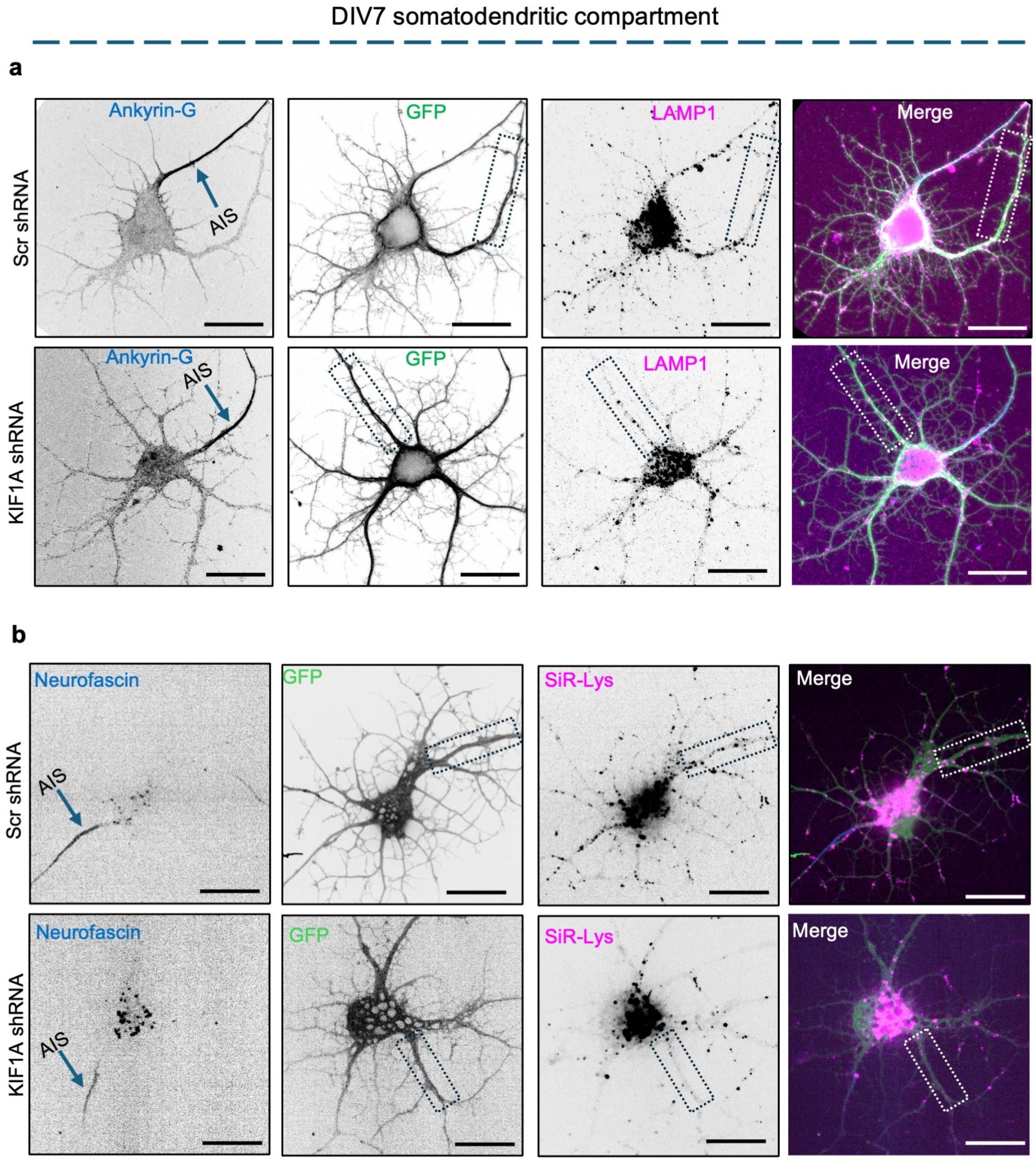

bioRxiv preprint doi: https://doi.org/10.64898/2026.10.29.762862; this version posted October 30, 2026. The copyright holder for this preprint (which was not certified by peer review) is the author/funder, who has granted bioRxiv a license to display the preprint in perpetuity. It is made available under a CC-BY 4.0 International license.

Figure S2. KIF1A depletion reduces lysosomal vesicle localization in the dendrites and axons of mature rat hippocampal neurons

a, Confocal fluorescence microscopy of the somatodendritic compartment of DIV7 rat hippocampal neurons expressing Scr shRNA or KIF1A shRNA along with GFP, fixed, permeabilize, and immunostained for ankyrin-G and LAMP1. The dashed outlines indicate the dendrites shown in Fig. 2a. Single-channel images are shown in grayscale, and merged images in color with ankyrin-G in blue, GFP in green, and LAMP1 in magenta. Scale bars: 20 μm.

b, Confocal live-cell imaging of the somatodendritic compartment of DIV7 rat hippocampal neurons expressing Scr shRNA or KIF1A shRNA along with GFP, stained with SiR-Lys and labeled for neurofascin to identify the AIS. The dashed outlines indicate the dendrites shown in Fig. 2e. Single-channel images are shown in grayscale, and merged images in color with neurofascin in blue, GFP in green, and SiR-Lys in magenta. Scale bars: 20 μm.

bioRxiv preprint doi: https://doi.org/10.64898/2026.10.29.762862; this version posted October 30, 2026. The copyright holder for this preprint (which was not certified by peer review) is the author/funder, who has granted bioRxiv a license to display the preprint in perpetuity. It is made available under aCC-BY 4.0 International license.

Figure S3

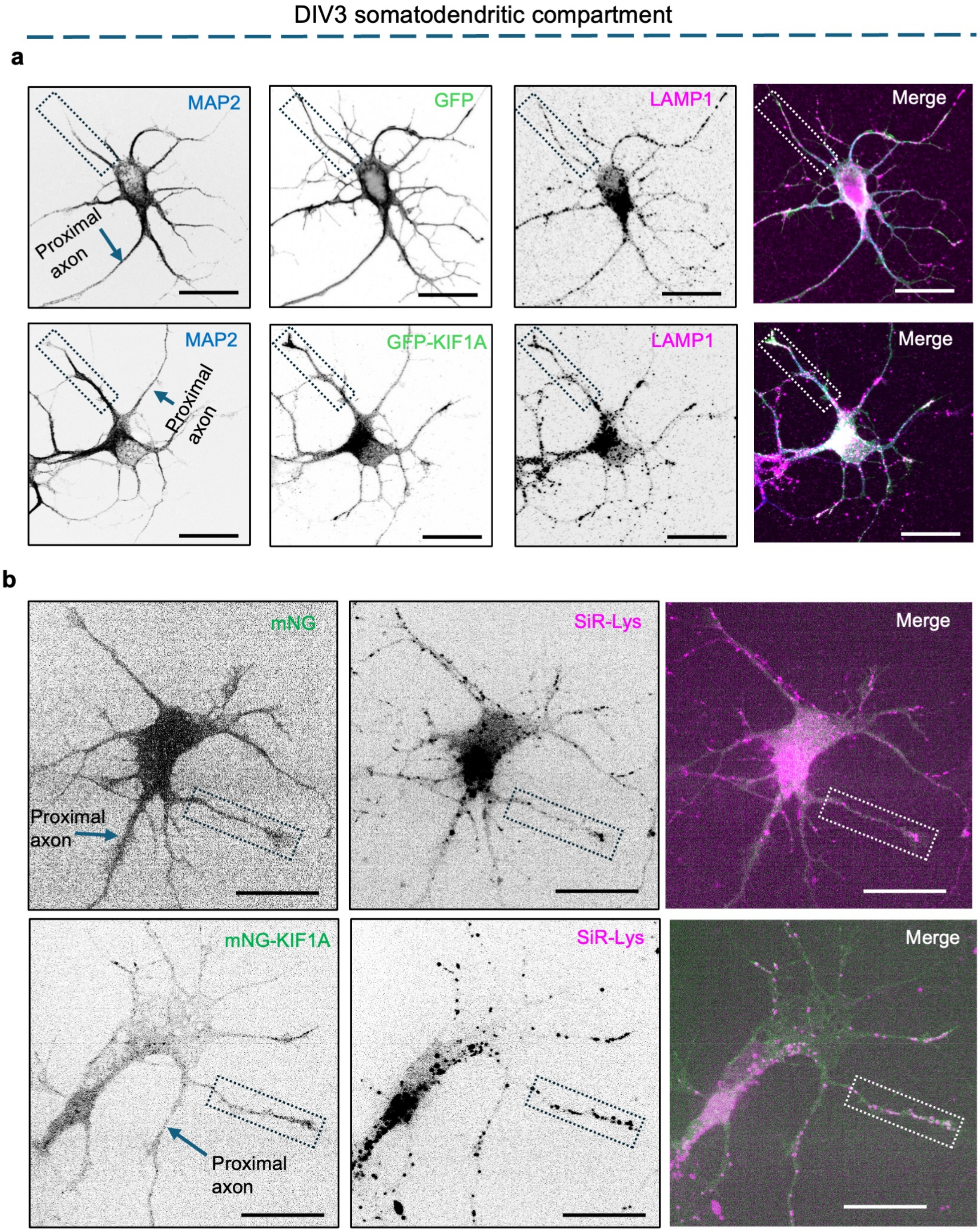

bioRxiv preprint doi: https://doi.org/10.64898/2026.10.29.762862; this version posted October 30, 2026. The copyright holder for this preprint (which was not certified by peer review) is the author/funder, who has granted bioRxiv a license to display the preprint in perpetuity. It is made available under a CC-BY 4.0 International license.

Figure S3. KIF1A expression promotes lysosomal vesicle accumulation in the dendrites and axons of immature rat hippocampal neurons

a, Confocal fluorescence microscopy of the somatodendritic compartment of DIV3 rat hippocampal neurons expressing GFP or GFP-KIF1A, fixed, permeabilized, and immunostained for MAP2 and LAMP1. The dashed outlines indicate the dendrite tips shown in Fig. 3a. Single-channel images are shown in grayscale, and merged images in color with MAP2 in blue, GFP or GFP-KIF1A in green, and LAMP1 in magenta. Scale bars: 20 μm.

b, Confocal live-cell imaging of the somatodendritic compartment of DIV3 rat hippocampal neurons expressing mNG or mNG-KIF1A and stained with SiR-Lys. The dashed outlines indicate the dendrite tips shown in Fig. 3e. Single-channel images are shown in grayscale, and merged images in color with mNG or mNG-KIF1A in green and SiR-Lys in magenta. Scale bars: 20 μm.

bioRxiv preprint doi: https://doi.org/10.64898/2026.10.29.762862; this version posted October 30, 2026. The copyright holder for this preprint (which was not certified by peer review) is the author/funder, who has granted bioRxiv a license to display the preprint in perpetuity. It is made available under a CC-BY 4.0 International license.

Figure S4

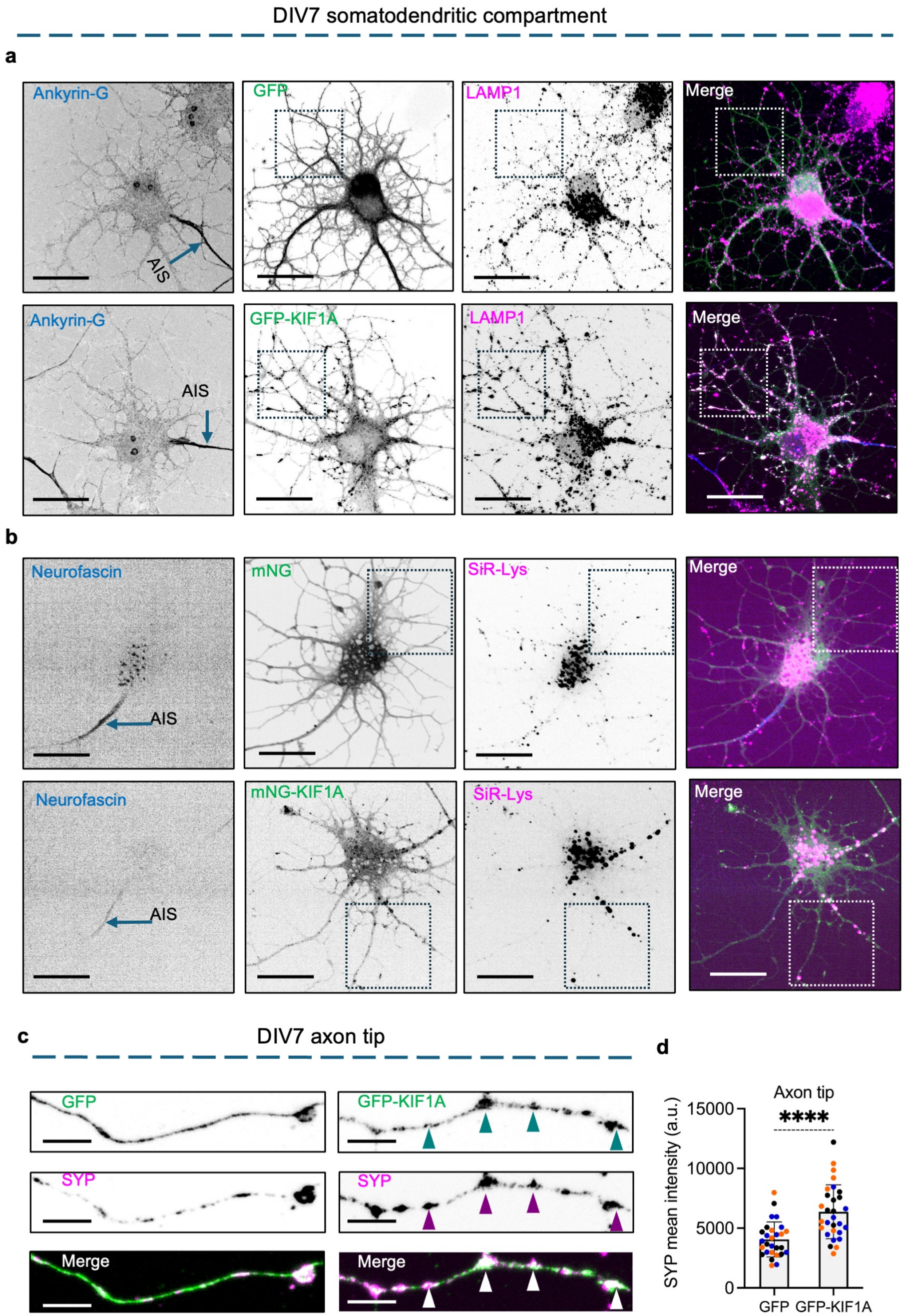

bioRxiv preprint doi: https://doi.org/10.64898/2026.10.29.762862; this version posted October 30, 2026. The copyright holder for this preprint (which was not certified by peer review) is the author/funder, who has granted bioRxiv a license to display the preprint in perpetuity. It is made available under a CC-BY 4.0 International license.

Figure S4. In mature rat hippocampal neurons, KIF1A expression increases accumulation of less degradative lysosomes and synaptophysin at the axon tips

a, Confocal fluorescence microscopy of the somatodendritic compartment of DIV7 rat hippocampal neurons expressing GFP or GFP-KIF1A, fixed, permeabilized, and immunostained for ankyrin-G and LAMP1. The dashed outlines indicate the dendritic regions shown in Fig. 4a. Single-channel images are shown in grayscale, and merged images in color with ankyrin-G in blue, GFP or GFP-KIF1A in green, and LAMP1 in magenta. Scale bars: 20 μm.

b, Confocal live-cell imaging of the somatodendritic compartment of DIV7 rat hippocampal neurons expressing mNG or mNG-KIF1A, stained with SiR-Lys and labeled for neurofascin to identify the AIS. The dashed outlines indicate the dendritic regions shown in Fig. 4e. Single-channel images are shown in grayscale, and merged images in color with neurofascin in blue, mNG or mNG-KIF1A in green, and SiR-Lys in magenta. Scale bars: 20 μm.

c, Confocal fluorescence microscopy of axon tips from DIV7 rat hippocampal neurons expressing GFP or GFP-KIF1A, fixed, permeabilized, and immunostained for synaptophysin (SYP). Single-channel images are shown in grayscale, and merged images in color with GFP or GFP-KIF1A in green and SYP in magenta. Arrowheads indicate representative GFP-KIF1A- and SYP-positive structures. Scale bars: 5 μm.

d, Quantification showing that GFP-KIF1A expression significantly increases SYP intensity at the axon tip. The graph shows individual data points and mean ± SD from 28–29 neurons per condition obtained from three independent cultures. Statistical significance was calculated by two-tailed unpaired Welch's t-test. ****$P < 0.0001$.

bioRxiv preprint doi: https://doi.org/10.64898/2026.10.29.762862; this version posted October 30, 2026. The copyright holder for this preprint (which was not certified by peer review) is the author/funder, who has granted bioRxiv a license to display the preprint in perpetuity. It is made available under a CC-BY 4.0 International license.

Figure S5

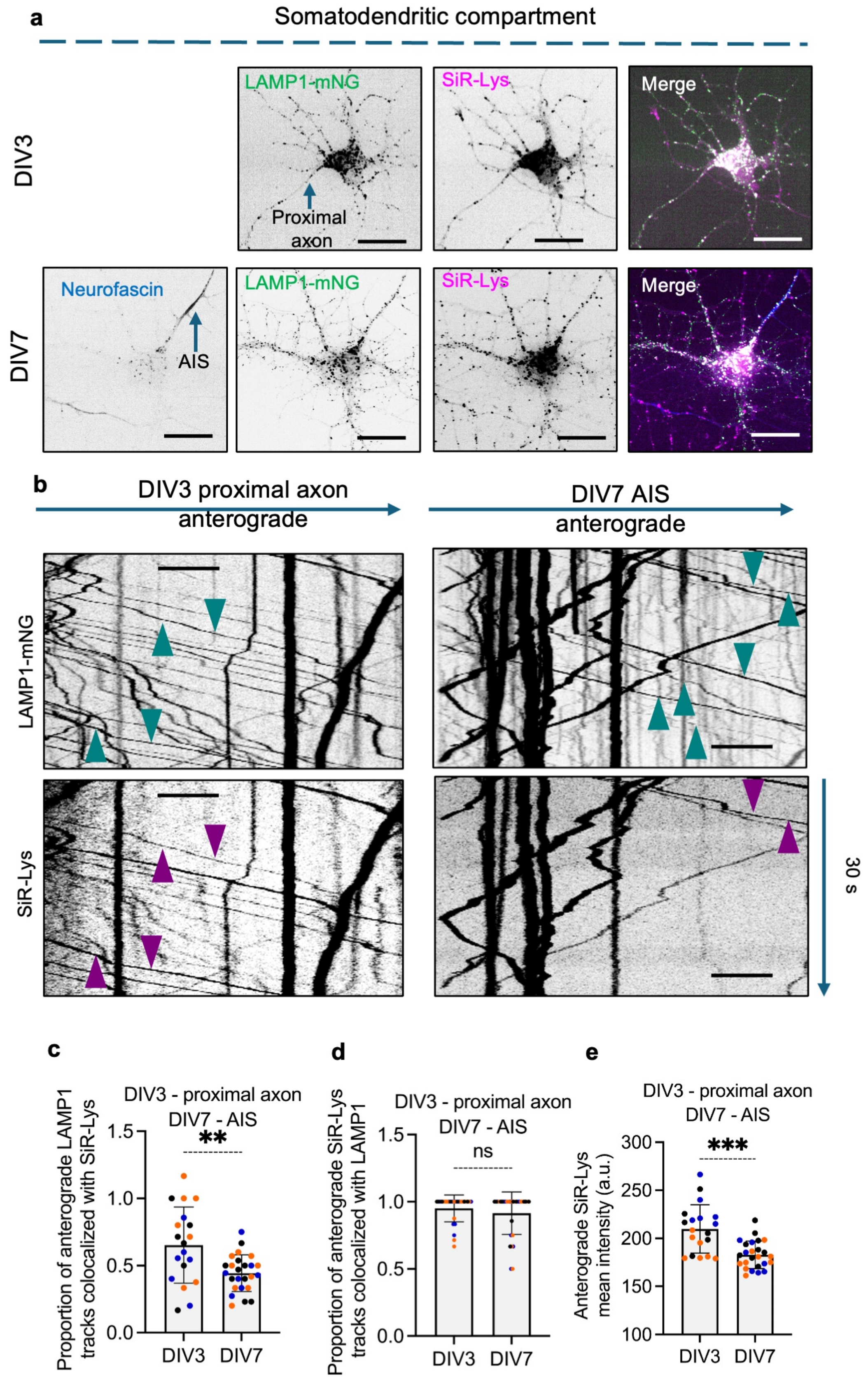

bioRxiv preprint doi: https://doi.org/10.64898/2026.10.29.762862; this version posted October 30, 2026. The copyright holder for this preprint (which was not certified by peer review) is the author/funder, who has granted bioRxiv a license to display the preprint in perpetuity. It is made available under a CC-BY 4.0 International license.

Figure S5. Neuronal maturation reduces the anterograde transport of degradative LAMP1-positive lysosomal vesicles

a, Confocal live-cell imaging of the somatodendritic compartments of DIV3 and DIV7 rat hippocampal neurons expressing LAMP1-mNeonGreen (LAMP1-mNG) and stained with SiR-Lys. Neurofascin labeling identifies the AIS in DIV7 neurons. Single-channel images are shown in grayscale, and merged images in color with LAMP1-mNG in green, SiR-Lys in magenta, and neurofascin in blue where shown. Scale bars: 20 μm.

b, Kymographs generated from live videos of the proximal axon of DIV3 neurons and the AIS of DIV7 neurons expressing LAMP1-mNG and stained with SiR-Lys. Teal arrowheads indicate representative LAMP1-mNG-positive anterograde tracks, and magenta arrowheads indicate SiR-Lys-positive anterograde tracks. Scale bars: 5 μm.

c, Quantification showing that the proportion of anterograde LAMP1-mNG-positive tracks colocalizing with SiR-Lys is significantly lower at the AIS of DIV7 neurons than in the proximal axon of DIV3 neurons. The graph shows individual data points and mean ± SD from 19–25 neurons per condition obtained from three independent cultures. Statistical significance was calculated by two-tailed Mann–Whitney test. $^{**}P < 0.01$

d, Quantification showing the proportion of anterograde SiR-Lys-positive tracks colocalizing with LAMP1-mNG in DIV3 and DIV7 neurons. The proportion is close to one in both developmental stages and is not significantly different between DIV3 and DIV7 neurons. The graph shows individual data points and mean ± SD from 19–25 neurons per condition obtained from three independent cultures. Statistical significance was calculated by two-tailed Mann–Whitney test. ns, not significant.

e, Quantification showing that anterograde SiR-Lys tracks moving through the AIS of DIV7 neurons are significantly fainter compared to those moving anterogradely through the proximal axon of DIV3 neurons. The graph shows individual data points and mean ± SD from 19–25 neurons per condition obtained from three independent cultures. Statistical significance was calculated by two-tailed Welch's t-test. $^{***}P < 0.001$.

bioRxiv preprint doi: https://doi.org/10.64898/2026.10.29.762862; this version posted October 30, 2026. The copyright holder for this preprint (which was not certified by peer review) is the author/funder, who has granted bioRxiv a license to display the preprint in perpetuity. It is made available under a CC-BY 4.0 International license.

Figure S6

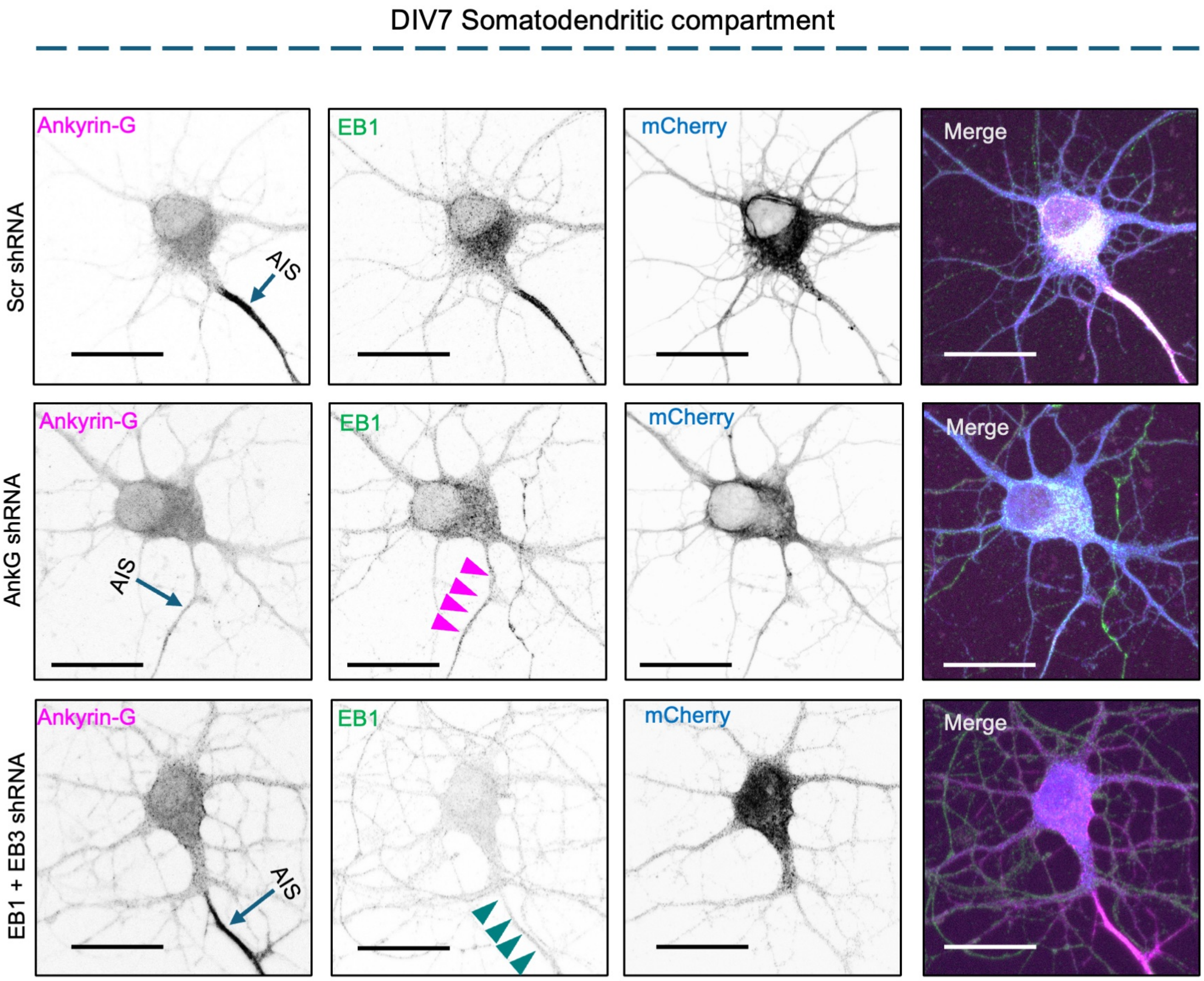

bioRxiv preprint doi: https://doi.org/10.64898/2026.10.29.762862; this version posted October 30, 2026. The copyright holder for this preprint (which was not certified by peer review) is the author/funder, who has granted bioRxiv a license to display the preprint in perpetuity. It is made available under a CC-BY 4.0 International license.

Figure S6. Ankyrin-G and EB1/EB3 depletion differentially alter AIS organization

Confocal fluorescence microscopy of the somatodendritic compartment of DIV7 rat hippocampal neurons expressing Scr shRNA, AnkG shRNA or EB1+EB3 shRNA along with mCherry, fixed, permeabilized, and immunostained for ankyrin-G and EB1. Single-channel images are shown in grayscale, and merged images in color with ankyrin-G in magenta, EB1 in green and mCherry in blue. The AIS is indicated. AnkG depletion reduces AnkG staining and is accompanied by loss of EB1 localization (magenta arrowheads), whereas upon EB1+EB3 depletion discernible ankyrin-G staining remains at the AIS but EB1 staining is markedly reduced (teal arrowheads). Scale bars: 20 μm.

bioRxiv preprint doi: https://doi.org/10.64898/2026.10.29.762862; this version posted October 30, 2026. The copyright holder for this preprint (which was not certified by peer review) is the author/funder, who has granted bioRxiv a license to display the preprint in perpetuity. It is made available under a CC-BY 4.0 International license.

Figure S7

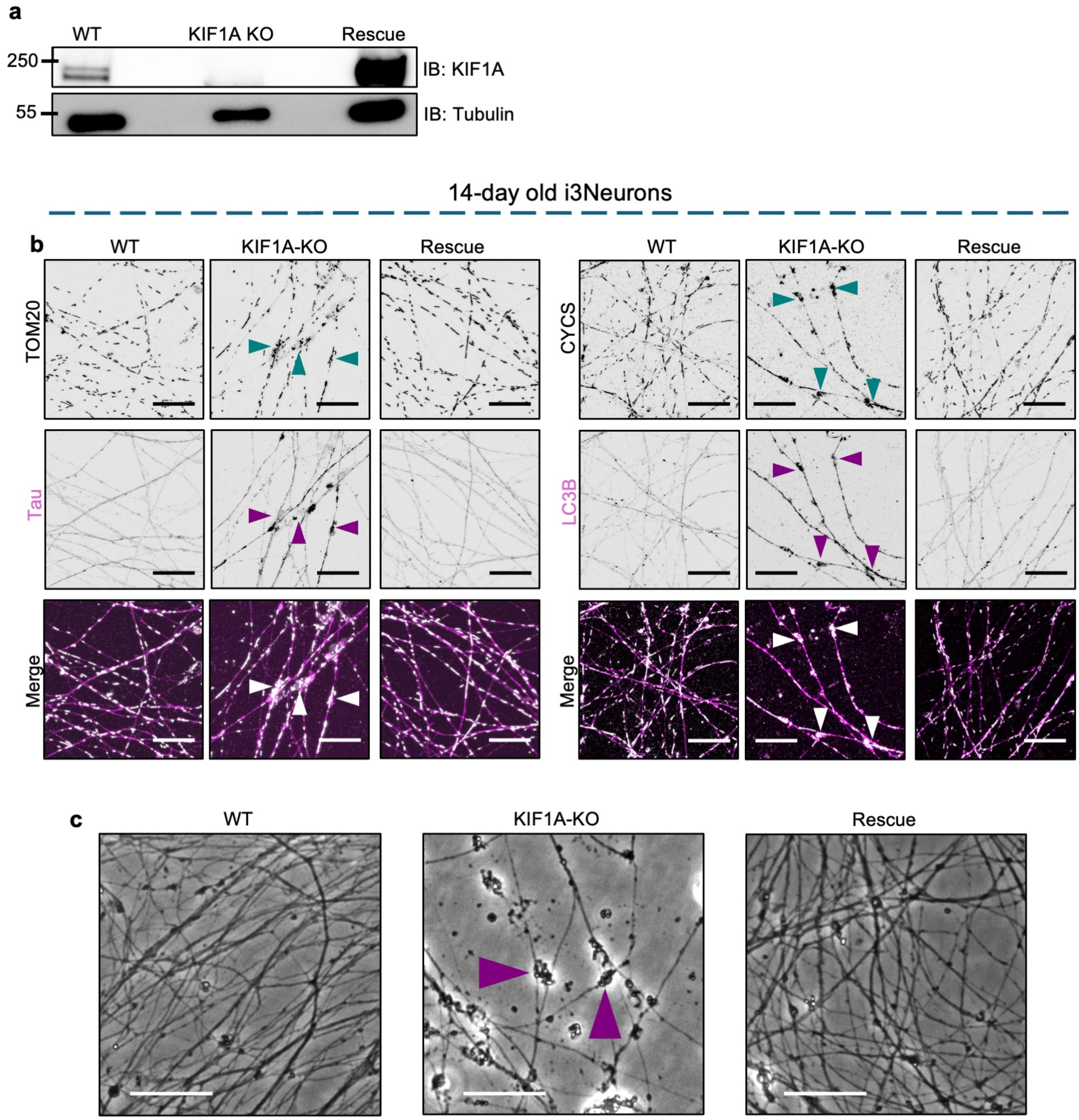

bioRxiv preprint doi: https://doi.org/10.64898/2026.10.29.762862; this version posted October 30, 2026. The copyright holder for this preprint (which was not certified by peer review) is the author/funder, who has granted bioRxiv a license to display the preprint in perpetuity. It is made available under a CC-BY 4.0 International license.

Figure S7. Validation of KIF1A knockout and rescue and associated axonal defects in human i3Neurons

a, Immunoblot analysis of lysates from WT, KIF1A-KO, and KIF1A-KO i3Neurons rescued with human KIF1A (Rescue), showing loss of KIF1A expression in KIF1A-KO neurons and restoration following rescue. Tubulin was used as a loading control. Molecular masses are indicated in kilodaltons.

b, Confocal fluorescence microscopy of primarily axonal fields from 14-day-old WT, KIF1A-KO, and rescued i3Neurons. Left – neurons immunostained for the mitochondrial protein TOM20 and the axonal marker Tau. Single-channel images are shown in grayscale, and merged images in color with TOM20 in white and Tau in magenta. Right – neurons immunostained for cytochrome c (CYCS) and the autophagosome marker LC3B. Single-channel images are shown in grayscale, and merged images in color with CYCS in white and LC3 in magenta. Green arrowheads indicate enlarged axonal regions containing accumulated mitochondria and magenta arrowheads indicate corresponding accumulation of Tau and LC3B-positive structures in KIF1A-KO neurons. Scale bars: 20 μm.

c, Representative transmitted-light images of 14-day-old WT, KIF1A-KO, and rescued i3Neurons. Arrowheads indicate prominent neurite swellings in KIF1A-KO neurons that are reduced following KIF1A rescue. Scale bars: 200 μm.

bioRxiv preprint doi: https://doi.org/10.64898/2026.10.29.762862; this version posted October 30, 2026. The copyright holder for this preprint (which was not certified by peer review) is the author/funder, who has granted bioRxiv a license to display the preprint in perpetuity. It is made available under a CC-BY 4.0 International license.

Supplementary Video 1. mNG-KIF1A and SiR-Lys movement through DIV3 proximal axon
Live-cell spinning-disk fluorescence microscopy of the proximal axon of DIV3 rat hippocampal neurons expressing mNG or mNG-KIF1A and stained with SiR-Lys. mNG or mNG-KIF1A is shown in green and SiR-Lys in magenta. Arrowheads indicate representative anterograde moving vesicles.

Supplementary Video 2. mNG-KIF1A and SiR-Lys movement through DIV7 AIS
Live-cell spinning-disk fluorescence microscopy of the AIS of DIV7 rat hippocampal neurons expressing mNG or mNG-KIF1A and stained with SiR-Lys. mNG or mNG-KIF1A is shown in green and SiR-Lys in magenta. White arrowheads indicate representative anterograde moving SiR-Lys vesicles and green arrowheads indicate representative anterograde moving mNG-KIF1A puncta not colocalized with SiR-Lys.

Supplementary Video 3. Comparison of LAMP1-mNG and SiR-Lys co-movement
Live-cell spinning-disk fluorescence microscopy comparing LAMP1-mNG and SiR-Lys movement through the proximal axon of DIV3 neurons and the AIS of DIV7 neurons. LAMP1-mNG is shown in green and SiR-Lys in magenta. White arrowheads indicate representative anterograde moving LAMP1-mNG and SiR-Lys colocalized vesicles and green arrowheads indicate representative anterograde moving LAMP1-mNG puncta not colocalized with SiR-Lys.

Supplementary Video 4. Effect of AnkG/EB shRNA on mNG-KIF1A and SiR-Lys co-movement
Live-cell spinning-disk fluorescence microscopy of the AIS of DIV7 rat hippocampal neurons expressing Scr shRNA, AnkG shRNA, or EB1+EB3 shRNA together with mNG-KIF1A and stained with SiR-Lys. mNG-KIF1A is shown in green and SiR-Lys in magenta. White arrowheads indicate representative anterograde moving SiR-Lys vesicles and green arrowheads indicate representative anterograde moving mNG-KIF1A puncta not colocalized with SiR-Lys.

Supplementary Video 5. Effect of T258M mutation on mNG-KIF1A and SiR-Lys co-movement
Live-cell spinning-disk fluorescence microscopy of the AIS of DIV7 rat hippocampal neurons expressing mNG, mNG-KIF1A-WT, or mNG-KIF1A-T258M and stained with SiR-Lys. mNG or mNG-KIF1A is shown in green and SiR-Lys in magenta. Arrowheads indicate representative anterograde moving vesicles.
